# Adverse events and deterioration reported by participants in the PACE trial of therapies for chronic fatigue syndrome

**DOI:** 10.1016/j.jpsychores.2014.04.002

**Published:** 2014-07

**Authors:** Dominic Dougall, Anthony Johnson, Kimberley Goldsmith, Michael Sharpe, Brian Angus, Trudie Chalder, Peter White

**Affiliations:** a East London Foundation NHS Trust, London, UK; b MRC Clinical Trials Unit at UCL, London, UK; c Biostatistics Department, Institute of Psychiatry, King's College London, UK; d Psychological Medicine Research, Department of Psychiatry, University of Oxford, Oxford, UK; e Nuffield Department of Medicine, University of Oxford, UK; f Academic Department of Psychological Medicine, King's College London, UK; g Wolfson Institute of Preventive Medicine, Barts and the London School of Medicine and Dentistry, Queen Mary University of London, London, UK

**Keywords:** Adverse events, Body mass index, Chronic fatigue syndrome, Depression, Medically unexplained symptoms

## Abstract

**Objective:**

Adverse events (AEs) are health related events, reported by participants in clinical trials. We describe AEs in the PACE trial of treatments for chronic fatigue syndrome (CFS) and baseline characteristics associated with them.

**Methods:**

AEs were recorded on three occasions over one year in 641 participants. We compared the numbers and nature of AEs between treatment arms of specialist medical care (SMC) alone, or SMC supplemented by adaptive pacing therapy (APT), cognitive behaviour therapy (CBT) or graded exercise therapy (GET). We examined associations with baseline measures by binary logistic regression analyses, and compared the proportions of participants who deteriorated by clinically important amounts.

**Results:**

Serious adverse events and reactions were infrequent. Non-serious adverse events were common; the median (quartiles) number was 4 (2, 8) per participant, with no significant differences between treatments (*P* = .47). A greater number of NSAEs were associated with recruitment centre, and baseline physical symptom count, body mass index, and depressive disorder. Physical function deteriorated in 39 (25%) participants after APT, 15 (9%) after CBT, 18 (11%) after GET, and 28 (18%) after SMC (*P* < .001), with no significant differences in worsening fatigue.

**Conclusions:**

The numbers of adverse events did not differ significantly between trial treatments, but physical deterioration occurred most often after APT. The reporting of non-serious adverse events may reflect the nature of the illness rather than the effect of treatments. Differences between centres suggest that both standardisation of ascertainment methods and training are important when collecting adverse event data.

## Highlights

• This study shows the distribution of adverse events in a trial of treatments for CFS.
• Non-serious adverse events were common, but no difference between treatments.
• Non-serious adverse events were more related to ill different than treatments.
• Deterioration in physical function was more likely after adaptive pacing therapy.
• Differences between centres may be related to different ascertainment methods.

## Introduction

Clinical trials frequently attribute health problems that arise during a trial to the intervention. But, when health problems typically remit and relapse, the attribution of all new health problems to the intervention may be misleading. This study aims to explore this issue in patients with chronic fatigue syndrome (CFS) who participated in a treatment trial.

Adverse events reported by participants in clinical trials of treatments may be considered to be clinically serious or not, and to be reactions to trial treatments or not. Few studies have examined the associations and predictions of adverse events in trials. Several trials have suggested a relationship between the reporting of adverse events and negative affect; anxiety [1], depression [2] and neuroticism [3]. Females and introverted participants of phase 1 medical trials are more likely to report adverse events than males and extroverts [4]. Physical symptoms at baseline predicted having a treatment related adverse reaction in an antidepressant controlled trial [5]. As well as this small literature regarding adverse events in trials, there are well established associations between reporting physical symptoms, outside of trials, and both mood disorders [6–10] and symptom burden [11].

Chronic fatigue syndrome (CFS) is characterised by long-standing disabling fatigue and other symptoms that have no alternative medical or psychiatric explanation [12]. Its nosological status and aetiology are uncertain [13]. CFS is associated with functional somatic syndromes such as irritable bowel syndrome and fibromyalgia [14]. Treatments recommended by the National Institute of Healthcare and Clinical Excellence (NICE) include cognitive behaviour therapy (CBT) and graded exercise therapy (GET) [15], but patient organisations have expressed concern about their efficacy and safety [16].

The PACE trial was a four arm randomised trial, which was designed to compare three therapies each added to specialist medical care (SMC) against SMC alone to determine both efficacy and safety [17]. The trial found that two therapies, CBT and GET, were more effective than adaptive pacing therapy (APT), when any of these therapies were added to SMC, and were more effective than SMC alone [17]. Whilst CBT and GET were designed to be rehabilitative, the goal of APT was to optimise adaptation to the illness by planning and pacing activities to avoid or reduce fatigue [17]. The trial measures of safety included systematic assessments of adverse events (AEs), which occur uncommonly in trials of behavioural interventions [18]. We have already reported that there were few serious adverse events (SAEs) and even fewer serious adverse reactions (SARs), the numbers of which did not differ significantly across treatment arms [17]. We have also reported various measures of deterioration, but not whether there are any differences across treatment arms in the proportions of participants who deteriorated in the two primary outcomes by a clinically important amount [17]. This paper reports the more commonly reported non-serious adverse events (NSAEs), compares their frequency between treatment arms, and also identifies baseline factors associated with reporting larger numbers of NSAEs [17,19]. On the basis of the previous literature, we hypothesised that NSAEs would be associated with female sex, a larger number of physical symptoms at baseline, and both depressive and anxiety disorders present at baseline. To our knowledge there has been no previous study examining associations of NSAEs in a trial of treatments for CFS or functional somatic syndromes.

## Methods

### Outline of the PACE trial

This report uses data from the PACE trial, relevant aspects of which are described; more comprehensive accounts are available in the protocol [19], and the primary paper [17]. The PACE trial recruited 641 patients from secondary care clinics with a diagnosis of CFS, using the Oxford criteria, which require six or more months of disabling fatigue, with fatigue being the principal symptom, and no alternative, explanatory diagnosis [20]. Participants were randomly allocated to one of four treatment arms consisting of specialist medical care (SMC) alone or SMC with one of APT, CBT or GET. Randomisation to the four treatment arms was stratified by centre, co-morbid depressive disorder, and different CFS and myalgic encephalomyelitis (ME) criteria [12,21]. Following randomisation, participants received up to 15 sessions of therapy (if allocated to a therapy arm) and at least 3 sessions of SMC.

All consecutive new outpatients from six secondary care CFS clinics in England and Scotland with a clinical diagnosis of CFS were clinically assessed for eligibility and, if they agreed, were screened by a research assistant (RA) for eligibility and consent for the trial. RAs were either nurses or psychologists, who were independent of clinical staff, but were not masked to treatment arms, this being impractical to achieve. There was only one RA per centre, but over the trial period, RAs left and were replaced in some centres. Recruitment commenced in March 2005 and was completed by Nov 2008. Follow-up was up to one year from randomisation.

Inclusion criteria were meeting the Oxford research diagnostic criteria for CFS [20], a Chalder Fatigue Questionnaire binary score of 6 or more [22], a SF36 physical function sub-scale score of 65 or less [23] and age at least 18 years old. Exclusion criteria were a significant risk of self-harm, being considered by the RA to be unable to participate in the trial, participation in the PACE trial being inappropriate for clinical needs, and patients who had previously attended a PACE centre specialist fatigue clinic and received a course of PACE trial consistent treatment [19].

The Structured Clinical Interview for DSM-IV was administered by the RA, after appropriate training, and used to assess psychiatric comorbidity and psychiatric exclusions [24]. Further baseline information collected included demographic details, current membership of a local or national ME self-help group, and body mass index (BMI). Additional self-report questionnaires included the Chronic Disease Self-Efficacy measure [25], physical symptoms (Patient Health Questionnaire; PHQ-15) [26], Cognitive Behavioural Responses Questionnaire (CBRQ) [27], Jenkins sleep scale of subjective sleep problems [28], and the Hospital Anxiety and Depression Scale (HADS) [29]. Further assessments consisted of the International (CDC) criteria for CFS [12], the London criteria for myalgic encephalomyelitis [21] and the presence or absence of fibromyalgia [30].

### Assessment of adverse events

Follow-up assessment interviews were conducted by the RA at each centre on three occasions: 12, 24 and 52 weeks after randomisation. At each of these time points the RA asked participants whether any new events or illnesses had taken place since the last assessment including any events for which the participant visited the GP or hospital department, or took medication [19]. AEs were also recorded by treating specialist doctors and therapists if spontaneously reported to them during the trial. An AE was defined as ‘any clinical change, disease or disorder experienced by the participant during their participation in the trial, whether or not considered related to the use of treatments being studied in the trial’ [19]. We did not examine inter-rater reliability between RAs since we did not foresee variability in these assessments.

AEs included: (a) any new co-morbid medical conditions reported, if not previously reported at baseline, (b) any events for which the participant consulted their GP or other medical advisor or took medication, and (c) any other events that might have affected the health status of the participant (e.g. increased work stress). Examples of NSAEs included a cold (which had not caused serious disability), an eye infection, or the experience of new pain (if not previously reported as a symptom of the participant's CFS). If in doubt, the RA was encouraged to contact the GP for both an update of all visits to the surgery since the last research session and a list of any medications prescribed. The RA also took note of any new events recorded in the clinic notes by the SMC doctor at these sessions or reports thereof from the treating specialist doctor or therapist.

Two consultant physicians and a consultant liaison psychiatrist, all experienced in CFS, were appointed as independent scrutineers and were masked to the participants' allocated treatment group. They determined whether each AE was serious or non-serious. A serious adverse event (SAE) was an event that resulted in one of the following outcomes: a) death, b) threat to life (i.e., an immediate, not hypothetical, risk of death at the time of the event), c) required hospitalisation except for elective treatment of a pre-existing condition, d) increased severity and persistent disability, defined as: (i) severe, i.e. significant deterioration in the participant's ability to carry out their important activities of daily living (e.g. employed person no longer able to work, caregiver no longer able to give care, ambulant participant becoming bed bound); and (ii) symptom and disability persistent, i.e. of at least 4 weeks continuous duration, e) any other important medical condition which, though not included in the above, might require medical or surgical intervention to prevent one of the outcomes listed, and f) any episode of deliberate self-harm. For any AE established as serious, the scrutineers were unmasked to treatment allocation to establish whether or not the event was a serious adverse reaction (SAR). A serious adverse reaction was considered to be a reaction to one of the supplementary therapies or a drug prescribed as part of SMC [19]. All those judged as definitely, probably, or possibly related were considered to be SARs.

A non-serious adverse event (NSAE) was any health event, which was not categorised as an SAE or SAR. Each NSAE was ascribed to the appropriate body system (gastroenterological, neurological, etc.) independently by two senior medical clinicians (one a consultant infectious diseases physician, the other a consultant liaison psychiatrist; both experienced in CFS), who were different from the independent scrutineers. NSAEs attributed to CFS (i.e. considered to be a symptom of CFS) were put into a separate category since there is no consensually agreed body system for CFS, and because of specific interest in these symptoms. Differences in clinicians' ratings were resolved by discussion until consensus was reached. To summarise, adverse events were any new health related event reported by the participant in any context. These were independently judged as serious adverse events, using an a priori guideline of seriousness, and as serious adverse reactions if independently judged to be a reaction to a trial intervention.

### Statistical analysis

Firstly, the frequencies of NSAEs reported by the participant were compared between treatment arms and centres by chi squared tests. Because there were individual differences between participants in the duration of follow-up (depending on drop-outs) we calculated the number of NSAEs per person year of follow-up, and compared these across treatment arms. The distribution of NSAEs across trial participants was non-normal, and attempts to normalise the distribution by transformation were unsuccessful, so we used non-parametric comparisons, such as Kruskal–Wallis tests. We then compared the proportions of participants with NSAEs attributed to different body systems across treatment arms, with a chi squared test.

A Poisson regression model was attempted with the frequency of NSAEs per participant, but this did not provide an adequate fit to the data due to the variance exceeding the mean (extra-Poisson variation); a negative binomial model was uninformative. Instead we used a median split (4/5) of the numbers of NSAEs, determined by analysis of the frequency of NSAEs per participant, as well as CFS related NSAEs per participant (0/1) in order to examine univariate associations with baseline characteristics. There were no informative independent studies of non-serious adverse events to guide us.

Secondly, univariate analyses of associations between NSAEs and other variables were conducted, with continuous variables transformed when not normally distributed. The chi-squared test was used for categorical variables. The t-test was used for continuous normally distributed variables.

Thirdly, all associated univariate variables, significant at *P* ≤ .1, were entered into a multivariate binary logistic regression model for all NSAEs, followed by a separate regression analysis for NSAEs attributed to CFS. Age, sex and treatment arm were also entered into all models. We also modelled those with one or more NSAEs versus those without any, using a logistic regression analysis to establish characteristic differences between these two groups of patients.

In order to provide further checks on the relatedness of adverse events in general to trial treatments, we compared both serious adverse events and reactions, and the numbers of participants in each treatment group who reported being “much worse” or “very much worse” in their overall health at 52 weeks after randomisation [31]. We also compared the numbers of participants in each treatment arm who had deteriorated by more than a clinically important difference (at least 2 points on the fatigue questionnaire and/or at least 8 points on physical function, which represented 0.5 of a standard deviation of baseline outcome measures) between randomisation and 52 weeks later [17]. We then examined the number of NSAEs in those who had deteriorated by either of the latter measures. We analysed the data using SPSS v18 and v22.

As a post-hoc analysis, in order to better understand the differences in NSAE counts between centres, we stratified centres into three groups: low (3 centres), medium (2 centres) and high (1 centre) numbers of NSAEs per participant. Using these strata, we undertook a one way analysis of variance (ANOVA) of those continuous variables that showed statistically significant differences by NSAE count on univariate analyses. We examined linear trends across centre strata.

## Results

Most (77%) participants were female, with only 7% from ethnic minorities. The mean (SD) age of participants was 38 (12) years. Approximately half of participants were educated to A-level or degree level standard (Appendix A, Table A). One participant withdrew their consent after participation, leaving 640 in the analysis.

**Fig. 1:**
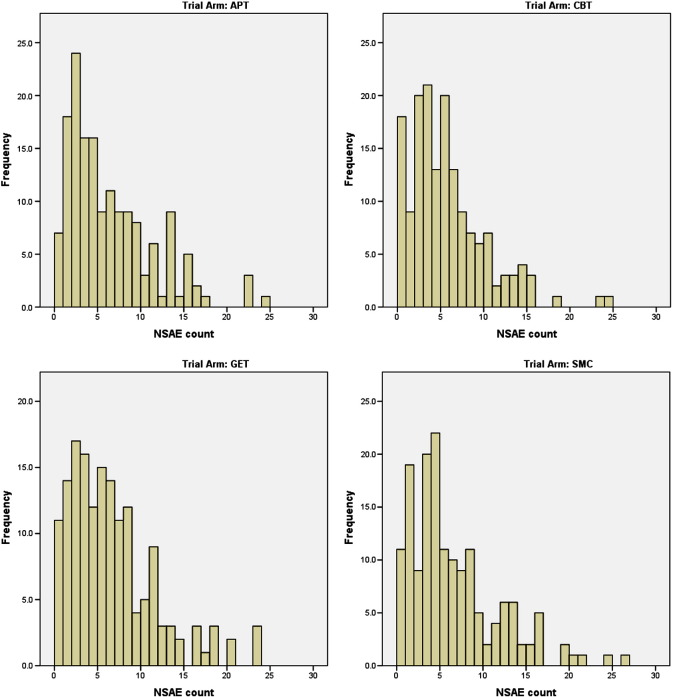
Histograms of non-serious adverse events by treatment arm.

The median (quartiles) number of reported NSAEs per participant per annum was 4 (2, 8), with no significant difference between treatment arms (Kruskal–Wallis test *P* = .47) (Table 1). This median was used to divide the sample into those with lower (≤ 4) and higher (≥ 5) numbers of NSAEs for the purpose of binary logistic regression analyses. The number (%) of trial arm participants with more than the median number of NSAEs varied from 78 (49%), in those allocated to CBT, to 90 (56%) in those allocated to GET (X^2^ = 2.34, 3 df, *P* < .51) (Table 1). Fig. 1 shows that the distributions of NSAE counts had similar patterns across the treatment groups, but with more participants having no NSAEs in the CBT group (post-hoc comparison of CBT participants versus all others combined: 6% versus 11%, chi-squared = 4.65, 1 df, *P* = .03).

A highly statistically significant difference in the reporting of NSAEs between trial centres was found (*P* < .001). This varied from a median of 4 in one centre to 10 in another centre (Appendix A, Table B).

### Types of NSAE

NSAEs affecting eyes, ears, nose and throat were reported by 54% of participants, with 46% of participants reporting NSAEs attributed to CFS (Table 2). Smaller numbers of participants had gastrointestinal, psychiatric/psychological and musculoskeletal NSAEs (Table 2). Chi squared tests showed no statistically significant differences in any of the comparisons across the four treatment arms (Table 2).

### Univariate associations

There were no significant associations of NSAEs with socio-demographic characteristics (Appendix A, Table A). In contrast, there were significant associations between increased reporting of NSAEs and several baseline variables: the numbers of both physical symptoms in general and symptoms of CFS, higher BMI, worse physical function, avoidance due to embarrassment, more fatigue, and HADS depression (Table 3). The mean (standard deviation) BMI was 25.5 (4.97) for all 640 participants. Some 123 (19%) participants were morbidly obese (BMI ≥ 30). Significant associations were also found between reporting more NSAEs and having any psychiatric disorder, particularly depressive disorder, dysthymia, and major depressive disorder (Table 4). There were no significant associations with anxiety disorders.

### Multivariable binary logistic regression of NSAEs

When modelling all NSAEs, using a median split of 4 or less versus 5 or more, centre effects dominated the models, so we remodelled both with and without centre. Without centre, a larger number of NSAEs were associated with baseline CFS symptom count (odds ratio (OR, 95% CI) 1.12 (1.01, 1.24), *P* = .03), physical symptom count 1.04 (0.99, 1.08), *P* = .09, baseline current depressive disorder 1.47 (1.04, 2.07), *P* = .03, and log body mass index 2.55 (1.09, 5.96), *P* = .03. With centre included, depressive disorder was lost from the model, as was the number of physical symptoms, but CFS symptoms (*P* < .001) and BMI (*P* = .035) were retained. There was no significant interaction between centre and treatment arm when this interaction term was entered into the model, indicating no differential effects of centre on treatments.

The one factor associated with one or more versus no NSAEs was CFS symptom count (1.17 (0.99, 1.38), *P* = .065). Adding centre to the model retained CFS symptom count (*P* = .01) and added treatment arm, but only at a *P* value of .10.

### Multivariable binary logistic regression of CFS related NSAEs

CFS related NSAEs were associated with baseline depressive disorder (1.81 (1.29, 2.53), *P* = .001) and baseline CFS symptom count (1.13 (1.03, 1.24), *P* = .008). Adding centre replaced baseline depressive disorder with baseline major depressive episode (*P* = .03) and added physical symptom count (*P* = .09) as well as CFS symptom count (*P* = .006).

### Post-hoc exploration of centre effects on NSAE count

Seven of ten variables measured at baseline were significantly correlated in a linear trend with centres stratified by NSAE count in the ANOVA (Appendix A, Table B). However, the variation between mean scores per centre varied little, with the most significant different variables of Chalder fatigue, SF36, and PHQ-15 varying by 2, 6 and 2.8 points respectively. There were no significant correlations for age, sex, duration of illness, and embarrassment scores.

### Deterioration by other measures

Table 5 shows that there were no statistically significant differences between treatment arms in those who had an SAE, SAR, or in those who had deteriorated either by CGI score (of either “much” or “very much worse”). This was also the case for fatigue alone and fatigue and disability combined. Similarly, there were no significant differences across treatment arms between the median number of NSAEs in those who had deteriorated as measured by the global impression change score (*P* = .97) and those reporting deterioration in both primary outcomes of fatigue and physical disability (*P* = .16) between treatment groups. However, there was a significant difference in deterioration of physical function, across treatment arms, with a quarter of those who received APT deteriorating in this way, compared to 18% after SMC, and 11% and 9% after GET or CBT respectively.

## Discussion

There were no important differences between treatment arms in any of the adverse events, however they were measured or classified. Most importantly there was no evidence of more frequent adverse events after either CBT or GET. The factors associated with a higher number of NSAEs were the centre where the participant was seen, followed by the number of physical symptoms at baseline, having a depressive episode, and higher body mass index. Those variables associated with CFS related NSAEs were centre, CFS symptom count and a depressive episode. The common baseline associations in both models were centre, depressive disorder and physical symptom count. Those who received APT were most likely to deteriorate by a clinically important amount in physical function, with those in receipt of CBT being least likely to deteriorate.

The substantial variation in the frequency of the reporting of NSAEs between centres is our most unexpected finding, although variation between centres is not uncommon in multi-centre trials [32], and this did not influence treatment response [17]. We found statistically significant linear associations between centres stratified by NSAE counts and a number of baseline variables, but the differences between centres were small, and nothing like the size of differences in NSAE frequency across centres. This suggests that the large differences in NSAE numbers between centres are unlikely to be related to the small differences found between centres in baseline factors. Although the research assessments across centres were standardised and training was provided at the start of the trial, it might be that the differences were due to different methods of ascertainment. This apparent variation in recording NSAEs, despite a standard protocol for doing so, has important implications for recording adverse events in future trials.

Having more symptoms at baseline, particularly those associated with CFS, predicted subsequent NSAEs in general and also NSAEs attributed to CFS. This replicates previous work [5]. Higher symptom counts are associated with somatoform disorders in secondary care [33], and may reflect a general tendency to report symptoms, which is associated with, but also independent of, mood disorders [34]. The specificity of CFS symptoms at baseline being associated with NSAEs attributed to CFS suggests a specific tendency to report these symptoms, rather than a generic influence of reporting any symptom. It may also reflect the relapsing and remitting nature of CFS.

Our finding that a diagnosis of a depressive disorder at baseline predicted increased reporting of NSAEs is consistent with previous studies that found negative affect to be associated with NSAEs specifically [1,3], and somatic symptoms in general [6–10,35]. This association remained significant for both NSAEs as a whole and for CFS attributed NSAEs in one regression model. Unlike some previous studies, we did not find an association with anxiety, either with the HADS score or through the SCID interview. One other trial failed to find an association between anxiety and adverse events [5].

We found that a higher BMI was associated with NSAEs in general. This observation may have several explanations; obese people generally report more of both physical and mental health related problems [36], and our sample included 123 (19%) participants who were morbidly obese. We were not able to replicate a previous research finding that female participants are more likely to report adverse events [4].

The strengths of this paper are that it used data from a large trial from multiple centres. The assessment of NSAEs on three occasions improved sensitivity. The limitations include the difference in frequency of NSAE reporting between centres, implying variation in ascertainment, although controlling for centre did not significantly affect our main findings. We only measured deterioration using self-ratings, rather than objective measures. We were unable to model the full distribution of NSAEs, which may have limited the power of our regression models.

## Conclusions

We found that there were no important differences in any of the adverse events between treatment arms, and no excess associated with either CBT or GET. Clinically important deterioration occurred least often after CBT and GET; APT may be associated with more frequent deterioration in physical functioning. We also noted that the reporting of non-serious adverse events in a clinical trial of treatment for CFS varied by recruitment centre, perhaps related to the method of ascertainment. This important finding has implications for the design of future trials. Research assessors need clear manualised guidance on the various definitions of adverse events, and specific training and supervision in order to implement them. We also found that baseline symptom count, having a depressive disorder and BMI were significantly associated with a greater number of NSAEs, independently of the treatment arms. This has both research and clinical implications for clinicians running trials, particularly those including patients with CFS. Adverse events in trials may more accurately reflect fluctuations in a condition, rather than reactions to interventions.

## Conflicts of interests

PDW has done voluntary and paid consultancy work for the United Kingdom government and a reinsurance company. TC has received royalties from Sheldon Press and Constable and Robinson. MS has done voluntary and paid consultancy work for the United Kingdom government, has done consultancy work for an insurance company, and has received royalties from Oxford University Press. DD, ALJ, KG and BA declare that they have no conflicts of interests.

## Funding

Funding for the PACE trial was provided by the Medical Research Council, the Department of Health for England, the Scottish Chief Scientist Office (G0200434), and the Department for Work and Pensions. DD was funded by the East London Foundation NHS Trust. TC acknowledges support from the NIHR Biomedical Research Centre for Mental Health at the South London and Maudsley NHS Foundation Trust and Institute of Psychiatry, Kings College London.

## Figures and Tables

**Table A:** Sociodemographic characteristics: univariate comparisons

Number of reported adverse events	NSAE ≤ 4		NSAE > 4			
	N	%	N	%	X^2^	*P*
Total (N)	313	49	327	51		
Gender (1 df)						
Female (495)	237	76	258	79	0.92	.34
Male (145)	76	24	69	21		
Ethnicity (1 df)						
Other (40)	20	6	20	6	0.038	.84
White (595)	288	94	307	94		
Educational level (5 df)						
Minimal (24)	17	5	7	2	6.51	.26
GCSE/equivalent (110)	47	15	63	19		
A level/equivalent (127)	64	20	63	19		
Degree (198)	96	31	102	33		
Postgraduate (114)	57	18	57	17		
Other (67)	32	10	35	11		
ME group member (3 df)						
Local only (33)	16	5	17	5	4.3	.23
National only (54)	20	7	34	10		
Both (18)	11	4	7			
No group (535)	266	50	269	82		

	Mean	SD	Mean	SD	t	*P*
Age at randomisation (years)	38.4	11.6	38.3	12.1	0.014	.79

**Table B:** Variation of non-serious adverse events and baseline variables by centre (N %)

	A	B	C	D	E	F	*P* value
Median (quartiles) NSAE count	3 (1, 4)	3 (2, 5)	4 (2, 6)	7 (4, 10)	8 (4, 13)	10 (6, 15)	< .001*
NSAE ≤ 4 (313)	86 (78)	74 (69)	78 (58%)	38 (34)	32 (29)	5 (8)	
NSAE > 4 (327)	24 (22)	34 (31)	57 (42%)	75 (66)	79 (71)	58 (92)	< .0001#
Chalder fatigue	28.5 (3.6)¶	28.1 (3.4)	27.2 (4.1)	28.8 (3.6)	28.0 (3.9)	29.2 (3.5)	.005$
SF36 PF	39 (16)	40 (16)	41 (13)	36 (16)	35 (16)	37 (16)	.006
CFS symptoms	5.0 (1.7)	4.6 (1.7)	4.3 (1.8)	4.6 (1.8)	4.7 (1.8)	5.4 (1.8)	.01
HADS anxiety	7.7 (4.4)	7.3 (3.7)	8.1 (4.6)	7.9 (4.2)	8.4 (4.2)	9.3 (3.8)	.008
HADS depression	8.3 (3.5)	7.6 (3.2)	8.2 (3.8)	8.4 (4.0)	8.4 (4.0)	9.4 (4.2)	.02
PHQ-15	14.4 (3.9)	13.8 (4.3)	12.9 (4.4)	14.2 (4.2)	14.8 (5.2)	15.7 (5.5)	< .001
BMI	25.2 (4.7)	27.0 (5.5)	24.0 (5.5)	25.5 (4.7)	25.1 (5.0)	27.2 (5.3)	.04

*Kruskal–Wallis. #*χ*2 (5 df) = 129.4, *P* < .0001. NSAE = non-serious adverse events. SF36 PF = SF36 physical function sub-scale score. HADS = Hospital Anxiety Depression Scale. PHQ-15 = Patient Health Questionnaire. ¶Last seven lines show means (SD). $The comparisons were by one way ANOVA across the centre strata of low, medium and high NSAE counts, with linear test for trend.

**Table 1:** Non-serious adverse events (NSAEs) by treatment arm N (%)

Treatment N	APT	CBT	GET	SMC
	159	161	160	160
Participants with NSAEs	152 (96)	143 (89)	149 (93)	149 (93)⁎
Number of NSAEs	949	848	992	977
Median (quartiles) NSAEs per person-year	4 (2, 9)	4 (2, 7)	5 (2, 8)	4 (3, 8)#
N (%) > median number of NSAEs	81 (50)	78 (49)	90 (56)	79 (49)¶

APT = adaptive pacing therapy, CBT = cognitive behavioural therapy, GET = graded exercise therapy, SMC = standardised specialist medical care.

⁎ X^2^ = 5.61, 3 df, *P* = .13.

# Kruskal–Wallis test of differences between treatment arms *P* = .47.

¶ X^2^ = 2.34, 3 df, *P* = < .51.

**Table 2:** Numbers (%) of participants with one or more non-serious adverse events by body system

Body system	Trial arm				%	X^2^	*P*
	APT (159)	CBT (161)	GET (160)	SMC (160)			
Eyes & ENT	91 (57)	87 (54)	81 (51)	87 (54)	54	1.41	.70
CFS/ME/PVFS	73 (46)	66 (41)	79 (49)	76 (48)	46	2.50	.47
Gastrointestinal	59 (37)	57 (35)	53 (33)	67 (42)	37	2.84	.42
Psychol/psychiatric	57 (36)	56 (35)	47 (29)	52 (33)	33	1.78	.62
Musculoskeletal	56 (35)	47 (29)	53 (33)	41 (26)	31	4.07	.25
Obs/gynae/urinary	40 (25)	34 (21)	34 (21)	33 (21)	22	1.22	.75
Respiratory	34 (21)	30 (19)	36 (23)	49 (31)	22	7.15	.067
Dermatological	33 (21)	21 (13)	30 (19)	35 (22)	19	4.91	.18
Neurological	26 (16)	26 (16)	31 (19)	39 (24)	19	4.58	.21
Stressful events	18 (11)	17 (11)	26 (16)	19 (12)	13	2.87	.41
Cardiovascular	8 (5)	11 (17)	8 (5)	11 (7)	6	0.97	.81
Nutrient & blood	5 (3)	2 (1)	8 (5)	3 (2)	3	4.83	.18
Allergies	2 (1)	6 (4)	6 (4)	4 (3)	3	2.47	.48
Endocrine	8 (5)	4 (2)	1 (1)	3 (2)	3	6.74	.081
Miscellaneous	4 (3)	5 (3)	3 (2)	8 (5)	3	2.88	.41

The table gives the number (%) of participants having one or more non-serious adverse events during their participation in the trial, separated into individual body systems. APT = adaptive pacing therapy, CBT = cognitive behavioural therapy, GET = graded exercise therapy, SMC = standardised specialist medical care. ENT = ear, nose and throat. CFS = chronic fatigue syndrome, ME = myalgic encephalomyelitis, PVFS = post-viral fatigue syndrome.

**Table 3:** Univariate comparisons of baseline variables in those below & above median number of non-serious adverse events (NSAEs)

Variable	NSAE ≤ 4 (N 313)		NSAE > 4 (N 327)		t	*P*
	Mean	SD	Mean	SD		
CFS symptom count	4.4	1.8	4.9	1.8	3.87	< .001
PHQ-15	13.5	4.3	14.8	4.8	3.63	< .001
BMI	25.0	4.8	25.9	5.1	2.40	.02
SF36 PF	39.6	15.2	36.5	16.2	2.48	.01
CBRQ embarrassment	11.6	5.5	12.6	5.5	2.33	.02
Fatigue	27.8	3.7	28.5	3.8	2.15	.03
HADS depression	8.0	3.6	8.6	3.9	2.14	.03
HADS anxiety	7.7	4.2	8.3	4.3	1.80	.07
CBRQ all or nothing	13.6	3.8	14.1	3.8	1.57	.12
CBRQ catastrophising	7.6	3.4	8.0	3.3	1.54	.12
WSAS	26.9	6.3	27.6	6.3	1.39	.16
Jenkins sleep	11.9	4.8	12.4	4.8	1.23	.22
Self-efficacy	4.8	1.5	4.7	1.6	0.86	.39
CBRQ behaviour avoidance	18.8	4.9	19.2	5.2	0.56	.58
CBRQ symptom focusing	12.8	4.9	13.0	5.0	0.51	.61
CBRQ fear avoidance	15.2	4.0	15.1	3.9	0.19	.85
CBRQ damage	11.0	3.4	11.0	3.3	0.025	.98

CFS = chronic fatigue syndrome, PHQ = Patient Health Questionnaire, BMI = body mass index, SF36 PF = short form 36 item physical function, HADS = Hospital Anxiety Depression Scale, CBRQ = Cognitive Behavioural Responses Questionnaire, WSAS = Work and Social Adjustment Scale.

**Table 4:** Association of baseline diagnoses with number of NSAEs below and above median N (%)

	NSAE ≤ 4		NSAE > 4		X^2^	*P*
Total	313	49%	327	51%		
London ME case						
Met (329)	177	56	152	46	6.49	.011
Not met (311)	136	44	175	54		
All psychiatric diagnoses						
Present (299)	128	41	171	52	8.35	.004
None (341)	185	59	156	48		
All depressive disorders						
Present (213)	85	27	128	39	10.35	.001
None (427)	228	73	199	61		
Major depressive disorder						
Present (112)	44	14	68	21	5.03	.025
None (528)	269	86	259	79		
Minor depressive disorder						
Present (67)	28	9	39	12	1.52	.220
None (573)	285	91	288	88		
Dysthymic disorder						
Present (71)	24	8	47	14	7.29	.007
None (569)	289	92	280	86		
All anxiety disorders						
Present (202)	92	29	110	34	1.33	.250
None (438)	221	71	217	66		
Generalised anxiety disorder						
Present (132)	56	18	76	23	2.80	.094
None (508)	257	82	251	77		
Fibromyalgia						
Present (138)	69	22	69	21	0.07	.79
None (501)	244	78	257	79		
SAEs or SARs during follow-up						
Present (51)	20	6	31	9	2.08	.15
None (589)	293	94	296	91		

All depressive disorders included major and minor depressive disorders and dysthymia. All anxiety disorders included generalised anxiety disorder, panic disorders, phobias and post-traumatic stress disorders. SAEs + SARs = number of participants with one or more serious adverse events or reactions.

**Table 5:** Serious adverse events/reactions and deterioration by 52 weeks (N %)

Treatment (N)	APT 159	CBT 161	GET 160	SMC 160	Chi-sq	*P*
Serious adverse events	15 (9)	7 (4)	13 (8)	7 (4)	5.3	.15
Serious adverse reactions	2 (1)	3 (2)	2 (1)	2 (1)	0.3	.96
Physical function worse	39 (25)	15 (9)	18 (11)	28 (18)	17.2	.0007
Fatigue worse	21 (13)	14 (9)	11 (7)	22 (14)	5.8	.12
Function & fatigue worse	11 (7)	4 (2)	5 (3)	8 (5)	4.6	.21
Median (quartile) NSAEs in those worse	8 (4, 11)	3 (2, 5)	7 (4, 14)	4 (3, 12)		.16
CGI worse	10 (6)	9 (6)	10 (6)	14 (9)	1.5	.69
Median (quartile) NSAEs in those worse by CGI	6 (4, 8)	8 (2, 11)	6 (2, 17)	4 (3, 14)		.97

Physical function worse = SF36 sub-scale score 8 or more points' deterioration; fatigue worse = Chalder fatigue questionnaire score 2 or more points' deterioration, CGI = Clinical Global Impression change = “much” or “very much” worse. APT = adaptive pacing therapy, CBT = cognitive behavioural therapy, GET = graded exercise therapy, SMC = standardised specialist medical care.
